# Primary hyperaldosteronism with bilateral branch retinal vein occlusion as the initial presentation: A case report

**DOI:** 10.1097/MD.0000000000046990

**Published:** 2026-01-09

**Authors:** Xiwen Ji, Jie Mu, Zhaoyang Wang, Xiaohan Yang, Chunli Chen

**Affiliations:** a Beijing Tongren Eye Center, Beijing Tongren Hospital, Capital Medical University, Beijing, P. R. China; b Qian Xi Nan People’s Hospital, Guizhou, P. R. China.

**Keywords:** anti-VEGF, bilateral branch retinal vein occlusion, dexamethasone implant, hypertension, macular edema, primary hyperaldosteronism

## Abstract

**Rationale:**

To report a rare case of primary hyperaldosteronism initially presenting with bilateral branch retinal vein occlusion (BRVO).

**Patient concerns:**

Having a 17-year history of poorly controlled hypertension, a 62-year-old male patient suffered from recurrent macular edema secondary to bilateral BRVO.

**Diagnoses:**

Laboratory tests revealed elevated plasma aldosterone concentration, lowered direct renin concentration, and an augmented aldosterone-to-renin ratio, with no suppression of plasma aldosterone on the Captopril test (15.2 ng/dL to 16.1 ng/dL). Diagnosis of bilateral BRVO with recurrent macular edema was further confirmed by ocular imaging, which predominantly comprised optical coherence tomography and fluorescein angiography.

**Interventions:**

The patient received systemic antihypertensive therapy. Ocular treatment included multiple intravitreal anti-vascular endothelial growth factor injections and dexamethasone implants.

**Outcomes:**

In the aftermath of combined systemic and intravitreal therapy, blood pressure was controlled, and the condition remained acceptably stable throughout follow-up.

**Lessons:**

This case sufficiently underscored the significance of systemic screening in patients with BRVO, especially those with refractory hypertension. It has been proven to be effective when systemic and intravitreal therapy are integrated, and long-term management is essential for the clinical treatments.

## 1. Introduction

As the second most prevalent retinal vascular disease, retinal vein occlusion (RVO) serves as a paramount cause of vision loss.^[1]^ Retinal vein occlusion can be classified into 2 types, including central retinal vein occlusion (CRVO) and branch retinal vein occlusion (BRVO) in line with the region discrepancies.^[2]^ BRVO occurs more frequently than CRVO, and the incidence of BRVO yields an estimated ratio of 0.4%.^[3]^ Nonetheless, bilateral involvement is comparatively uncommon in BRVO. As evidently demonstrated by previous studies, 5% to 6% of eyes had bilateral BRVO at baseline and 10% developed fellow eye involvement.^[4]^ Treatment for BRVO is principally intended to address the complications like macular edema and prevent further vision deterioration. The prognosis of BRVO demonstrates dissimilar states. To be specific, some patients require fewer injections of anti-VEGF or corticosteroid medications, with longer intervals between injections and better visual prognosis. In contrast, others frequently experience recurrent macular edema, thereby necessitating more frequent anti-VEGF or corticosteroid injections, characterized by less time-consuming intervals between treatments and less favorable visual prognosis. It is evident that effective management of the latter group remains a pivotal clinical problem.

Proven risk factors for RVO encompass hypertension, diabetes mellitus and dyslipidemia.^[5,6]^ Nonetheless, these factors fail to throw light upon all occurrences of RVO. Primary hyperaldosteronism (PA) functions as a principal cause of secondary hypertension.^[7]^ In particular, the prevalence of PA has been reported to be 5% to 10% in BRVO.^[8–11]^ As already suggested by previous studies, PA can cause damage to cardiovascular and renal vascular endothelium.^[12,13]^ Notwithstanding the fact that several studies have reported ocular ischemic conditions in patients with PA,^[14,15]^ it remains rarely documented with reference to the cases of bilateral BRVO in these patients.

In this study, we describe a clinical case of PA initially presenting with bilateral BRVO.

## 2. Case description

**Figure 1.**
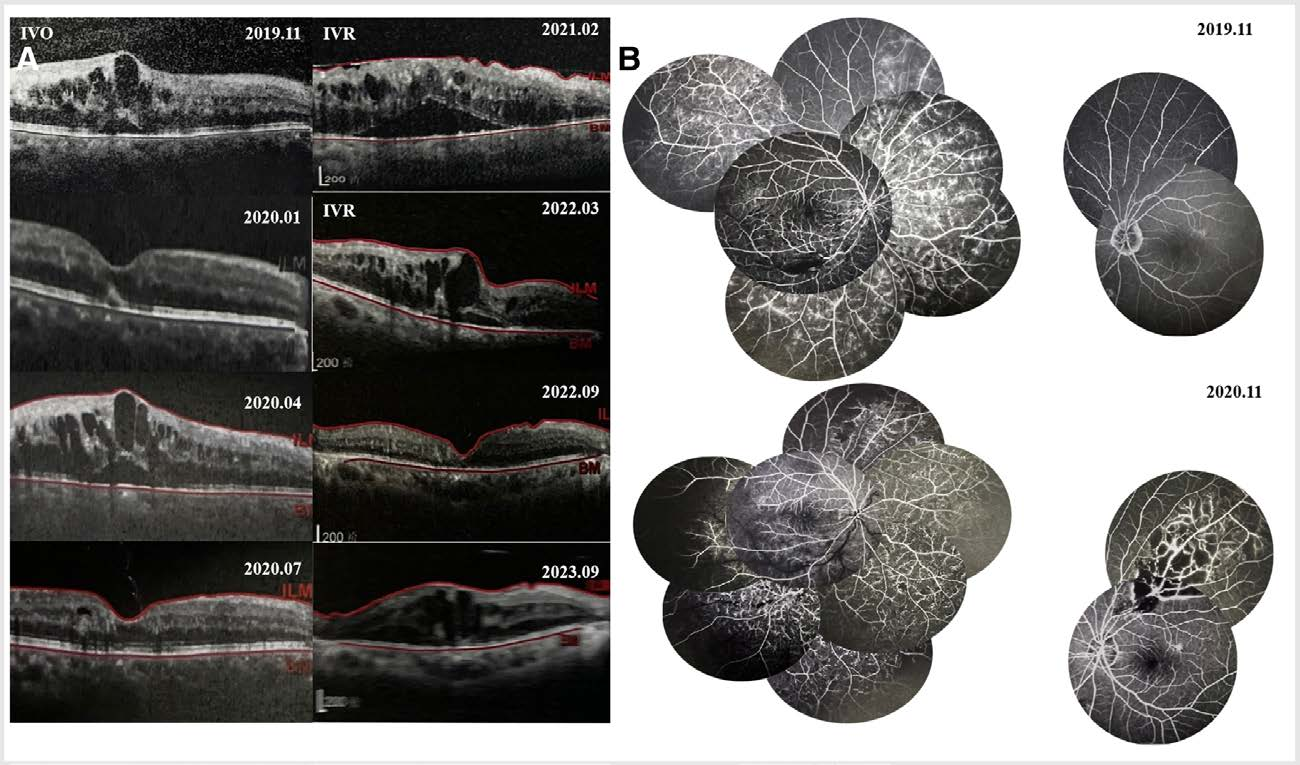
(A) The results of optical coherence tomography (OCT) from 2019 to 2023 showed the status of cystoid macular edema (CME) was unstable during the treatment in another hospital. (B) Fluorescein fundus angiography (FFA) showed that fundus non-perfusion area in 2020.11 increased in the right eye compared to the result in 2019.11 and his left eye fundus began to appear non-perfusion area in 2020.11.

The patient was a 62-year-old man who presented in 2023 for evaluation of a 4-year history of painless vision loss in his right eye. The patient was diagnosed with hypertension 17 years ago. Notwithstanding the employment of multifarious medications, the diastolic blood pressure has remained consistently between 100 mm Hg and 110 mm Hg and could not be reduced further. Four years ago, the patient was diagnosed with non-ischemic BRVO in the right eye at an external healthcare facility. Subsequent to a 1-year follow-up, the right eye progressed to ischemic BRVO, and the left eye developed BRVO as well. Severe recurrent macular edema, retinal hemorrhage, and ischemic changes have been observed in the right eye over the past 4 years. The patient had received over ten administrations of intravitreal anti- VEGF injections and was treated with intravitreal dexamethasone implant 3 times. Aside from that, he reported a history of multiple sessions of retinal photocoagulation therapy. We obtained the results of optical coherence tomography (OCT) from 2019 to 2023, which displayed the status of cystoid macular edema (CME) was unstable throughout the treatment in another hospital (Fig. 1A). Fluorescein fundus angiography (FFA) revealed a progressive enlargement of the fundus non-perfusion area in the right eye over a 1-year period. Apart from that, subsequent assessment illustrated the development of novel non-perfusion areas in the left eye.(Fig. 1B).

**Figure 2.**
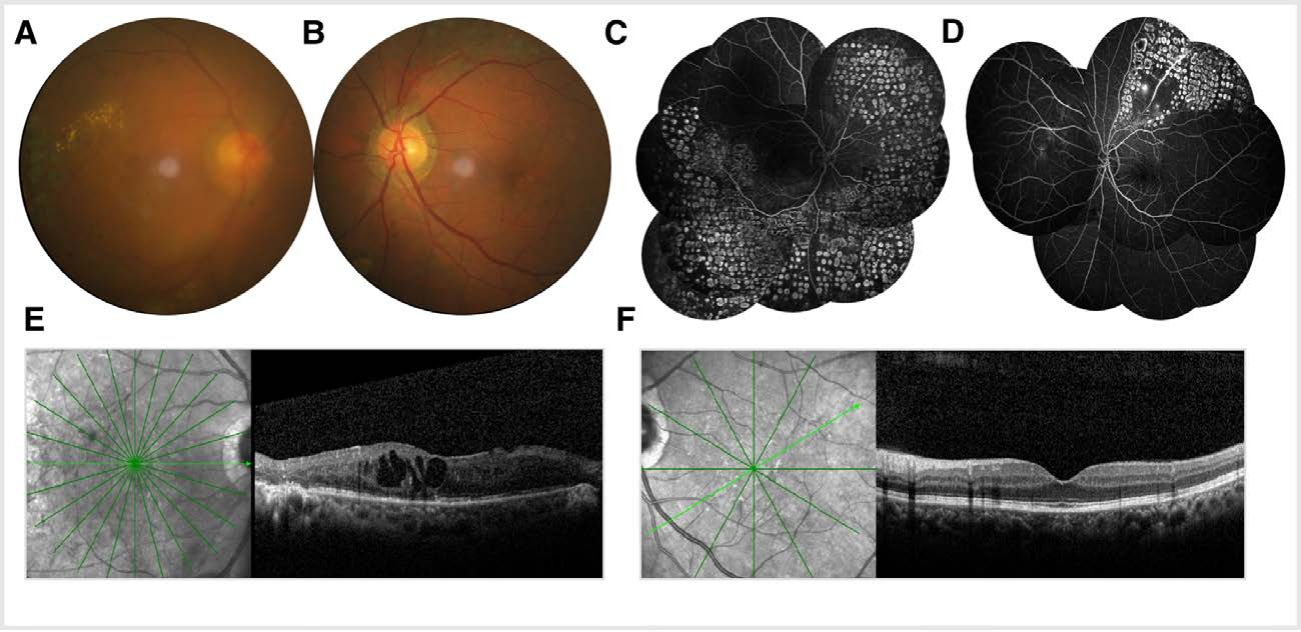
(A) In the right eye funduscopy, the optic disc was moderately clear with distinct margins. The macula exhibited edema, with a hazy appearance. Retinal hemorrhages were visible along the affected vein distribution. And there were laser photocoagulation scars scattered in the peripheral retina. (B) In the left eye funduscopy, the optic disc appeared well-defined and healthy. The macula showed only mild changes, with minimal signs of edema. And the retinal vasculature seemed relatively preserved. Meanwhile, laser photocoagulation scars were only presented in the superior temporal quadrant. (C) The fluorescein angiography showed hyperfluorescence due to optic disc leakage and multiple hypofluorescent lesions corresponding to hemorrhages and retinal ischemia in right eye. And regions of laser treatment could be clearly seen. (D) Laser-treated areas could be seen. (E) OCT showed CME in right eye. (F) OCT didn’t show CME in left eye.

As the physical examination revealed, the best corrected visual acuity of right and left eyes were 0.03 and 0.8, respectively and the intraocular pressure was 9 mm Hg in OD and 10mmHg in OS. Anterior segment evaluation illustrated no conspicuous abnormalities except for mild lens opacity. In the right eye funduscopy, the optic disc was moderately clear with distinct margins. The macula exhibited edema, with a hazy appearance. Retinal hemorrhages were visible along the affected vein distribution. Simultaneously, there were laser photocoagulation scars scattered in the peripheral retina (Fig. 2A). The left eye exhibited a well-defined optic disc, mild macular changes without noticeable edema, and relatively preserved retinal vasculature, with laser scars confined to the superotemporal quadrant (Fig. 2B). FFA of the right eye revealed hyperfluorescence as a consequence of optic disc leakage, throughout which multiple hypofluorescent areas corresponding to hemorrhages and retinal ischemia should also be taken into consideration. Concurrently, previous laser treatment zones were also conspicuous (Fig. 2C). The pattern in the right eye was more remarkable than that in the left eye. Sparse hypo-autofluorescent spots suggested laser-treated areas (Fig. 2D). Simultaneously, CME in the right eye was confirmed by OCT (Fig. 2E). Nonetheless, noticeable macular edema cannot be observed in his left eye (Fig. 2F). For this reason, the studied male patient was diagnosed as bilateral BRVO.

**Figure 3.**
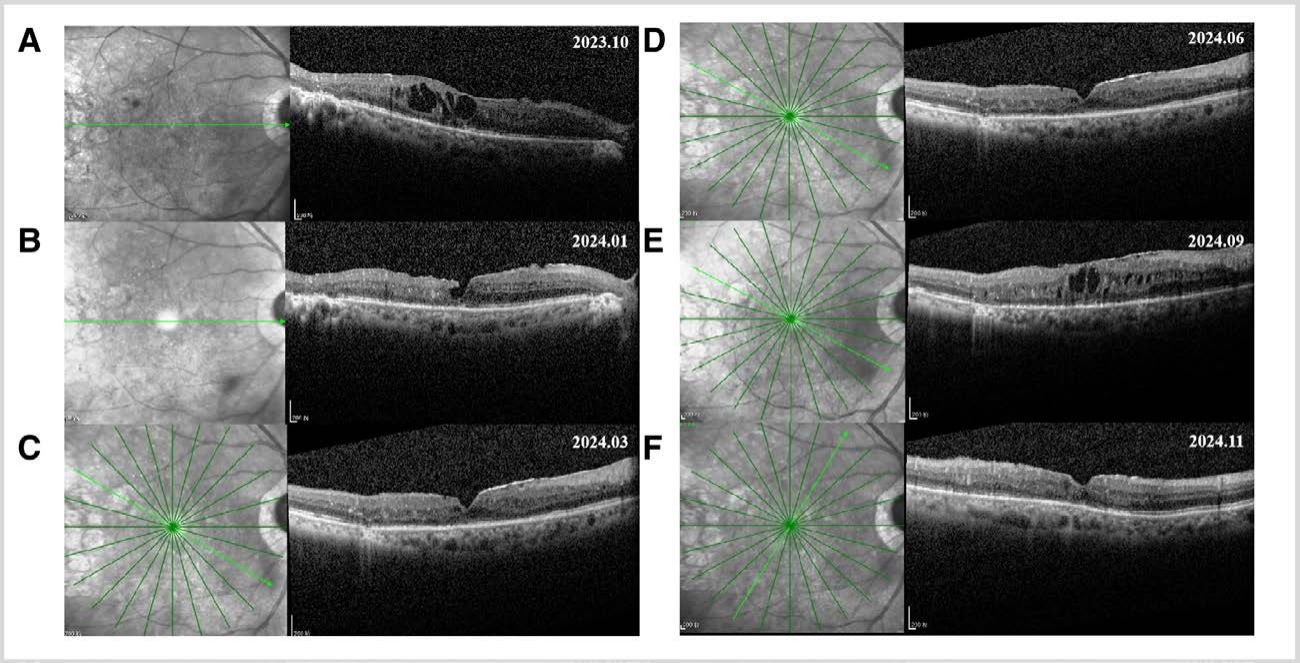
(A) OCT showed CME in right eye was still obvious in October 2023. (B) OCT didn’t show CME in right eye in January 2024. (C) OCT didn’t show CME in right eye in March 2024. (D) OCT didn’t show CME in right eye in June 2024. (E) OCT showed CME in right eye recurred in September 2023. (F) OCT didn’t show CME in right eye in November 2024.

On account of persistent CME and non-perfusion areas in the right eye, we treated him with intravitreal ranibizumab and micro-pulse laser to reduce CME in the right eye in September 2023 (Fig. 2E). Follow-up OCT in October 2023 suggested continuous CME (Fig. 3A). As a consequence, intravitreal ranibizumab and dexamethasone were jointly recommended for him. Simultaneously, he was treated with micro-pulse laser again.

It is particularly noteworthy that systemic screening for its etiology was carried out in consideration of the 5-year history of bilateral BRVO. Plasma aldosterone concentration (PAC) was 15.1ng/dL, which is above the normal range (5–10 ng/dL). Direct renin concentration was 3.7 mU/L, which is lower than the normal range (5–50 mU/L). The classic screening test of PA, aldosterone to renin ratio was 4.081, exceeding the cutoff value. Saline infusion test displayed PAC was 9.8 ng/dL after intravenous saline drip, which was recorded between 5–10 ng/dL. As illustrated by the captopril experimental findings, the level of plasma aldosterone before captopril was taken was 15.2 ng/dL, which was not more than the level of plasma aldosterone after captopril was taken, 16.1 ng/dL. Last but not least, a diagnosis of primary hyperaldosteronism (PA) was established, and the patient was started on spironolactone.

Follow-up examinations at 3, 5, 8, 11, and 13 months suggested ameliorated diastolic blood pressure control (consistently below 90 mm Hg) and fundus stability until June 2024 (Fig. 3B, 3C, 3D). Nonetheless, although the severity was milder compared to before starting spironolactone treatment, macular edema was observed again in the right eye during a follow-up in September 2024 (Fig. 3E). In September, we administered anti-VEGF treatment. Upon reexamination in November, the macular edema had resolved (Fig. 3F). For his left eye, he was treated with ranibizumab once. The patient was closely followed-up throughout the treatment, and the vision decline of both eyes wasn’t observed any longer. His visual acuity was 0.05 in the right eye and 0.8 in the left eye 1 year after the treatment.

## 3. Discussion

We have described a case of bilateral BRVO associated with PA. BRVO normally occurs at an arteriovenous crossing where the major artery and vein share a common adventitial sheath. Moreover, the artery typically passes anterior to the vein.^[16]^ Despite the fact that the precise pathological mechanism of BRVO remains ambiguous, a combination of haemodynamic changes, degenerative alteration of the vessel wall and blood hypercoagulability, including involvement of systemic and local risk factors, has been put forth in a systematic manner.^[17,18]^ This patient was 62 years old and diagnosed with secondary hypertension, both of which will give rise to the rigidity of arterial walls. On top of that, the mechanical compression of rigid arterial walls will narrow the venous lumen, ultimately bringing about turbulent blood flow at the A/V crossing,^[19]^ which serves as a crucial pathological mechanism of this patient. Vein occlusion can result in tissue hypoxia, which in turn leads to the release of vascular inflammatory factors such as VEGF. Serious complications can be triggered subsequent to the release of inflammatory factors and retinal hypoxia. It is paramount to mention that macular edema (ME) and neovascularization are the 2 most frequent complications of BRVO, which are also the 2 chief causes of vision loss. Retinal neovascularization could be observed in one third of untreated eyes.^[20]^ Meanwhile, one review revealed that 5% to 15% of BRVO eyes develop ME over 1 year.^[4]^ For the time being, these 2 complications are primarily targeted by ocular treatment of BRVO.

In line with the result of FFA, the NP area progressively enlarged in his right eye and began to appear in his left eye with the progression of the disease course, which suggested a progressive deterioration of retinal ischemia. In practical terms, standardization has not been adequately materialized with regard to the criteria for diagnosing ischemic BRVO. One research defined ischemic BRVO as retinal non-perfusion encompassing more than 5 disc diameters,^[21]^ while another defined that retinal ischemia covering at least 50% of the area for ischemic BRVO.^[22]^ Regardless of the specific criteria, expansion of retinal ischemia symbolizes a typical trend in the natural history of BRVO. As also revealed by prior research, 28.6% nonischemic BRVO turned to ischemic BRVO after 1 year following up.^[22]^ On that account, BRVO patients are strongly recommended to return for reviewing the development of their disease, which holds pivotal significance. To go it further, discovering predictive factors of ischemic development is significant to prevent any complications of ischemia.^[23]^ If we detect ischemia in time, we could intervene in its evolution.

Intravitreal anti-VEGF acts as an established treatment for macular edema in BRVO.^[24,25]^ As prior studies evidenced, anti-VEGF contributes to ameliorated central retinal sensitivity and lowered central retinal thickness for patients with BRVO in the long term.^[26,27]^ This patient had received anti-VEGF treatment several times. Nevertheless, not every administration had obvious clinical improvement. As a result, it is imperative to conduct more profound investigation, so as to establish clearer criteria for deciding whether to continue or alter anti-VEGF therapy in poorly responsive cases. Meanwhile, dexamethasone intravitreal implant could be employed to assist anti-VEGF therapy in the treatment of severe recurrent macular edema. Anti-VEGF and micro-pulse laser served as an integrated treatment for this patient at the initial stage, yet the macular edema persisted after 1 month. Hence, an integration of anti-VEGF and intravitreal dexamethasone was administered in a targeted manner. Follow-up examination 2 months later revealed resolution of the macular edema. When the efficacy of anti-VEGF monotherapy is undesirable,^[28]^ combined dexamethasone intravitreal implant can be taken into consideration owing to the fact that the occurrence of macular edema is bound up with inflammatory response, which may improve the prognosis of patients.^[29]^

There are a variety of systemic diseases and other ocular diseases associated with BRVO besides primary hypertension and diabetes. Diseases causing a hypercoagulable state of the blood are one of the etiology.^[30]^ Inflammatory diseases such as Behcet disease^[31]^ potentially result in retinal vascular inflammation, ultimately giving rise to venous occlusion. Hemopathy such as polycythemia vera could lead to BRVO, through raising blood viscosity and slowing venous blood flow.^[32]^ Metabolic disorders such as hyperhomocysteinemia^[33]^ and primary aldosteronism^[15]^ could also bring about BRVO. Vascular damage and hypertension induced by elevated homocysteine levels and aldosterone function as paramount factors in the pathogenesis of thrombosis and retinal vein occlusion. Simultaneously, elevated intraocular pressure in glaucoma patients may exert a certain influence on retinal venous outflow, eventually bringing about occlusion.^[34]^ The patient was diagnosed with primary hyperaldosteronism, complicated by BRVO, following a series of laboratory tests. His diastolic blood pressure was controlled in the aftermath of taking spironolactone, stabilizing below 90 mm Hg. If the patient had been diagnosed with PA earlier, his condition would have been controlled at an earlier stage. Therefore, screening test should be paid more attention.

PA serves as a contributor of BRVO primarily by inducing endothelial dysfunction. Other than the endothelial injury spawned from secondary hypertension, heightened aldosterone levels perse can impose direct and adverse influences on endothelial function. Aldosterone-induced endothelial dysfunction primarily involves 4 mechanisms: vascular inflammation, impaired vascular tone, vascular remodeling, and early atherosclerosis.^[35]^ In this case, the development of BRVO in this male patient was probably attributable to endothelial dysfunction induced by aldosterone.

Considering the diversity with respect to the causes of BRVO, our department has a standardized diagnosis and treatment protocol for patients with newly diagnosed BRVO. The first step is to identify the cause of BRVO by medical consultation and laboratory tests. If essential, the patient will be referred to the rheumatology and immunology department. The second point is to adopt appropriate treatment. Providing that the patient is diagnosed with BRVO for the first time, 3 anti-VEGF treatments should be administered first, followed by FFA examination to determine if there is an NP area and whether laser supplementation is needed. Assuming that macular edema was recurrent, FFA examination should be a top priority, which aimed at the determination of whether anti-VEGF combined with laser photocoagulation is a necessity.

We found only 2 case reports which described patients suffering with retinal vein occlusion and PA either.^[15,36]^ The deficiency of systematic screening for secondary hypertension in retinal vein occlusion patients may account for the inconclusive results in prior studies.

To sum up, we reported a case of an elderly patient who suffered from bilateral BRVO and PA. This case demonstrated PA might have contributed to the occurrence of BRVO. Nevertheless, more cases should be reported before exact conclusions can be drawn regarding the role of PA in the development of retinal vascular occlusive disorders.

## Author contributions

**Data curation:** Xiwen Ji, Jie Mu, Chunli Chen.

**Formal analysis:** Xiwen Ji.

**Funding acquisition:** Xiaohan Yang.

**Methodology:** Jie Mu.

**Supervision:** Zhaoyang Wang, Xiaohan Yang, Chunli Chen.

**Writing – original draft:** Xiwen Ji, Jie Mu.

**Writing – review & editing:** Zhaoyang Wang, Chunli Chen.
